# 1’-Acetoxychavicol acetate inhibits growth of human oral carcinoma xenograft in mice and potentiates cisplatin effect via proinflammatory microenvironment alterations

**DOI:** 10.1186/1472-6882-12-179

**Published:** 2012-10-09

**Authors:** Lionel LA In, Norhafiza M Arshad, Halijah Ibrahim, Mohamad Nurul Azmi, Khalijah Awang, Noor Hasima Nagoor

**Affiliations:** 1 Institute of Biological Science (Genetics and Molecular Biology), Faculty of Science, University of Malaya, Kuala Lumpur 50603, Malaysia; 2 Institute of Biological Science (Ecology and Biodiversity), Faculty of Science, University of Malaya, Kuala Lumpur 50603, Malaysia; 3 Centre for Natural Product Research and Drug Discovery (CENAR), Dept. of Chemistry, Faculty of Science, University Malaya, Kuala Lumpur 50603, Malaysia

**Keywords:** 1’-Acetoxychavicol acetate, *Alpinia conchigera*, NF-κB, CDDP, Oral Cancer, HSC-4

## Abstract

**Background:**

Oral cancers although preventable, possess a low five-year survival rate which has remained unchanged over the past three decades. In an attempt to find a more safe, affordable and effective treatment option, we describe here the use of 1’S-1’-acetoxychavicol acetate (ACA), a component of Malaysian ginger traditionally used for various medicinal purposes.

**Methods:**

Whether ACA can inhibit the growth of oral squamous cell carcinoma (SCC) cells alone or in combination with cisplatin (CDDP), was explored both *in vitro* using MTT assays and *in vivo* using *Nu/Nu* mice. Occurrence of apoptosis was assessed using PARP and DNA fragmentation assays, while the mode of action were elucidated through global expression profiling followed by Western blotting and IHC assays.

**Results:**

We found that ACA alone inhibited the growth of oral SCC cells, induced apoptosis and suppressed its migration rate, while minimally affecting HMEC normal cells. ACA further enhanced the cytotoxic effects of CDDP in a synergistic manner as suggested by combination index studies. We also found that ACA inhibited the constitutive activation of NF-κB through suppression of IKKα/β activation. Human oral tumor xenografts studies in mice revealed that ACA alone was as effective as CDDP in reducing tumor volume, and further potentiated CDDP effects when used in combination with minimal body weight loss. The effects of ACA also correlated with a down-regulation of NF-κB regulated gene (FasL and Bim), including proinflammatory (NF-κB and COX-2) and proliferative (cyclin D1) biomarkers in tumor tissue.

**Conclusion:**

Overall, our results suggest that ACA inhibits the growth of oral SCC and further potentiates the effect of standard CDDP treatment by modulation of proinflammatory microenvironment. The current preclinical data could form the basis for further clinical trials to improve the current standards for oral cancer care using this active component from the Malaysian ginger.

## Background

Oral cancers are malignancies arising from either tongue, lip, gingivae, palate, salivary glands, buccal mucosa or floor of the mouth [1], and accounts for an estimated 2.08% (263,900) of total cancer cases worldwide in 2011 [2]. About 90% of oral cancers are squamous carcinomas [3], with main treatment options that include surgery followed by radiotherapy and adjuvant chemotherapy [4]. Even though oral cancers are relatively preventable, diagnosed patients often face a low five-year survival rate of 58%, which has remained unchanged over the past three decades despite recent treatment advances [5]. Presently, platinum-based drugs such as cisplatin (CDDP), remains one of the most commonly used chemotherapeutic agents available for the treatment of advanced oral cancers [6]. While CDDP treatment often results in initial responses and disease stabilization, its long-term success is hindered by the development of drug resistance and dose-limiting toxicities through the occurrence of DNA cross-linking in surrounding non-cancerous cells [7]. Thus, there is an ongoing need for modified CDDP combination regimes that can ideally reduce overall dose-toxicity through chemo-sensitization of oral cancer cells.

Implications of inhibiting the nuclear factor kappa-B (NF-κB) pathway to sensitize cancer cells have been previously reported on various cell types including prostate epithelial cells and bladder cells [8-10]. NF-κB is a transcription factor which is constitutively present in the cytoplasm as an inactive heterotrimer consisting of p50, p52, p65 (RelA) and IκBα subunits. Upon activation by various cytokines and chemokines, IκBα undergoes phosphorylation and subsequent ubiquitination-dependant degradation, allowing NF-κB heterodimers to freely translocate and retain within the nucleus to promote transcription [11,12]. Overexpression of κB-regulated genes has been linked with most cancers, and can mediate events such as cellular transformation, proliferation, invasion, angigogenesis and metastasis [13]. Agents that suppress NF-κB activation are typically sought for as chemo-sensitizers since it regulates an array of genes governing the sensitivity of cells towards drugs such as glutathione S-transferase (GST), which is an enzyme involved in metal metabolism, whereby its overexpression has been linked to the resistance of cis-platinum drugs in SCCs [14].

The use of 1’S-1’-acetoxychavicol acetate (ACA), which is a phenylpropanoid naturally found within various Zingiberaceae family members, has been traditionally associated with a number of various medicinal properties including anti-ulceration [15], anti-allergic [16], anti-inflammatory and anti-cancer activities [17,18]. Previous studies have shown ACA to be associated with the production of intracellular reactive oxygen species (ROS) [19], inhibition of xanthane oxidase (XO) activity [20], inhibition of nitric oxide synthase (NOS) expression [21], inhibition of polyamine synthesis [22], induction of apoptosis via mitochondrial/Fas-mediated dual mechanism [19] and as a potential NF-κB inhibitor [23-25]. Even though the structure-activity relationship of ACA has been thoroughly studied [26], its intracellular molecular effects on downstream protein candidates involved in sensitization remain unidentified.

In this study, we investigated the role of ACA as a chemo-sensitizer on oral SCC cells and its combined effects with CDDP *in vivo* to produce an improved chemotherapeutic regime with increased efficacies at lower concentrations, which could hypothetically reduce the occurrence of dose-limiting toxicities.

## Methods

### Plant material

Rhizomes of *Alpinia conchigera* Griff were collected from Jeli province of Kelantan, East-coast of Peninsular Malaysia. The sample was identified by Prof. Dr. Halijah Ibrahim from the Institute of Biological Science, Division of Ecology and Biodiversity, Faculty of Science, University of Malaya. A voucher specimen (KL5049) was deposited in the Herbarium of Chemistry Department, Faculty of Science, University of Malaya.

### Reagents

NE-PER protein extraction kit and SuperSignal West Pico chemiluminescent substrate were purchased from Pierce (IL, USA). Suicide Track™ DNA ladder isolation kit, MTT reagent, propidium iodide (PI), mitomycin-C, Suicide Track^TM^ DNA ladder isolation kit and CDDP were obtained from EMD Chemicals Inc. (CA, USA). Primary NF-κB antibodies p65, IκB-α, IKK-α, IKK-β, histone H3 and β-actin were obtained from Santa Cruz Biotechnology (CA, USA). Antibodies against FasL, Bim, xIAP, poly-(ADP-ribose) polymerase (PARP), SignalStain^®^ Boost IHC detection reagents and IHC antibodies against NF-κB p65, IκBα, phospho-IKKα/β, COX-2, and cyclin D1 were obtained from Cell Signalling (MA, USA). RNeasy^® ^Plus Mini Kit was purchased from Qiagen (Germany), while LIVE/DEAD^®^ Viability/Cytotoxicity kit for mammalian cells was purchased from Molecular Probes, Invitrogen (NY, USA).

### Cell lines and culture conditions

Human oral squamous carcinoma cells (HSC-4) were obtained from Dr. Eswary Thirthagiri of the Cancer Research Initiative Foundation (CARIF, Malaysia), while human mammary epithelial cells (HMEC) (Lonza Inc., USA) were used as a normal cell controls. All cells were cultured as monolayers in Dulbecco’s Modified Eagle’s Medium (DMEM) supplemented with 10.0% (v/v) FBS, 100 U/ml penicillin and 100.0 μg/ml streptomycin, while HMEC cells were cultured in Mammary Epithelial Growth Medium (MEGM). All cultures were maintained in humidified incubator at 37°C in 5.0% CO_2_ and 95.0% air.

### MTT cell viability assay

The cytotoxic effect of ACA on HSC-4 and HMEC cells was determined by measuring MTT dye uptake and metabolism. ACA was dissolved in dimethyl sufoxide (DMSO) to a final concentration of 10.0 mM. Briefly, 2.0 x 10^4^ cells were treated in triplicates on 96-well plates in the presence or absence of ACA and/or in combination with CDDP at final concentrations of 5.0 μM to 80.0 μM up to 36 h. Final DMSO concentration in each experiment was maintained below 0.5% (v/v) to prevent solvent induced cytotoxicty. 20.0 μl of MTT dye reagent (5.0 mg/ml) was added to each well and cells were incubated in the dark at 37°C. After 2 h of incubation, media containing excess dye was aspirated and 200.0 μl of DMSO was added to dissolve purple formazon precipitates. A microtiter plate reader (Tecan Sunrise^®^, Switzerland) was used to detect absorbance at a test wavelength of 570 nm, with a reference wavelength of 650 nm.

### Live and dead assay

Assessment of cell viability upon treatment with ACA was accomplished using the LIVE/DEAD^®^ Viability/Cytotoxicity kit for mammalian cells according to manufacturer’s protocol. Cancer and normal cell lines were grown as monolayers on cover slips for 24 h and treated with ACA (15.0 μM) for 3 h and 6 h. Staining of cells were done using a dual fluorescence staining system consisting of 150.0 μl of both calcein-AM (2.0 μM) which emits green fluorescence when cleaved by intracellular esterases, and ethidium homodimer (EthD) (4.0 μM) which emits red fluorescence upon binding to nucleic acid in non-viable cells. Excitation and emission wavelengths of both fluoresceins were set at 494/517 nm for calcein-AM and 528/617 nm for EthD respectively. Visualization of samples was done using a Nikon Eclipse TS-100 fluorescence microscope (Nikon, Japan) under 100x magnification with dual pass filters for simultaneous viewing of both stains.

### Migration assay

The anti-migration effects of ACA were determined using the wound healing assay. HSC-4 cells were seeded in 6-well plates and allowed to form monolayers overnight. Growth medium was then changed to serum-free medium containing mitomycin-C and further incubated in 37°C for 2 h to halt proliferation of cells. Scratch wounds of equal size were introduced into the monolayer by a sterile pipette tip and cell debris generated from the scratch was washed away with 1x phosphate-buffered saline (PBS). Cells were treated with vehicle or IC_20_ ACA (10.0 μM) in serum-free medium for 24 h and microscopic images describing speed of wound closure was documented at various time intervals using an inverted fluorescence microscope, Nikon Eclipse TS-100 and analyzed using TScratch software, Version 1.0 (MathWorks Inc.).

### PARP cleavage assay

The occurrence of apoptosis was assessed based on the proteolytic cleavage of PARP by caspase 3. Briefly, cells (2.0 x 10^6^/mL) were treated with ACA (15.0 μM) and total proteins were extracted using the NE-PER^®^ nuclear and cytoplasmic extraction kit according to manufacturer’s protocol. Fractionation was done using SDS-PAGE and electro-transferred onto nitrocellulose membranes. Total proteins were incubated with rabbit anti-PARP antibodies and detected using an enhanced chemiluminescence reagent using x-ray films. Apoptosis was represented by cleavage of 116-kDa PARP into an 85-kDa product.

### DNA fragmentation assay

Cells were treated with ACA (15.0 μM) for 12 h and 24 h before harvesting, and total DNA was extracted from both untreated and treated cells using the Suicide Track^TM^ DNA Ladder isolation kit according to the manufacturer’s protocol. Extracted DNA was analysed on a 1.0% (w/v) agarose gel electrophoresis and stained with ethidium bromide. Fragmentation of DNA was observed under UV illumination and visualized using a gel documentation system (Alpha Inotech, USA).

### Microarray global gene expression analysis

To investigate changes brought upon by ACA in global gene expression, the Affymetrix GeneChip^®^ Human Gene 1.0 Sense Target (ST) Array (Affymetrix Inc., USA) was used according to manufacturer’s protocol. Briefly, total RNA from HSC-4 cells treated with ACA (15.0 μM) for 60 min and 120 min were extracted using the RNeasy^®^ Plus Mini Kit according to manufacturer’s protocol and analyzed under the Agilent 2100 Bioanalyzer (Agilent Technologies, CA, USA). RNA samples were then reverse transcribed, labelled and hybridized onto Affymetrix chips containing 764,885 probes representing and spanning across 28,869 human genes. Scanning of all arrays was done using the Affymetrix GeneChip^®^ Scanner (Affymetrix Inc., USA). Statistical and gene expression analysis of triplicate arrays were done using the GeneSpring^®^ GX version 10.0 (Agilent Technologies, CA, USA) software employing principle component analysis plots, *p*-value and fold-change thresholds.

### Western blot analysis

To determine levels of protein expression, cytoplasmic and nuclear extracts from HSC-4 cells treated with ACA at IC_50_ concentrations for 2 h and 4 h were prepared using the NE-PER^®^ nuclear and cytoplasmic extraction kit according to manufacturer’s protocol. Protein concentration was quantified and normalized using the Quick Start Bradford protein assay kit 2 (Bio-Rad, USA) according to manufacturer’s protocol. Fractionation of proteins were done using a 12.0% (v/v) SDS-PAGE and electrophoretically transferred to a 0.2 μm nitrocellulose membrane using the TransBlot SD Semi Dry Transfer Cell (Bio-Rad, USA). Blots were blocked and incubated with 13 primary antibodies: β-actin, histone H3, FasL, xIAP, Bim, p65, phospho-p65 (Ser536), IκBα, phospho-IκBα (Ser32/36), IKKα, phospho-IKKα (Thr23), IKKβ and phospho-IKKβ (Ser176) overnight at 4°C. Detection of bound antibodies were done using HRP-conjugated secondary antibodies, and visualized using the SuperSignal West Pico chemiluminescent substrate on x-ray films. Normalization of protein concentration was carried against β-actin and histone H3 proteins for cytoplasmic and nuclear components respectively. Relative intensities of all bands were quantified using ImageJ v1.43 analysis software (NIH, USA).

### Combination effects of ACA and CDDP

Assessment of synergistic drug combination treatments between ACA and CDDP were evaluated using MTT assays on HSC-4 cells as previously described [27]. A total of 2.0 x 10^4^ cells were plated in triplicates and treated with standalone ACA, standalone CDDP, and ACA in combination with CDDP at various concentration ratios for duration of 24 h and 48 h exposure. In groups where ACA were held constant, a sub-optimal IC_25_ of dose 5.0 μM was used, while for CDDP constant groups, a sub-optimal IC_25_ dose of 30.0 μg/ml was used. After incubation, 5.0 mg/ml MTT reagent was added into each well, incubated for 2 h in the dark at 37°C until a purple formazan precipitate was clearly visible and absorbance measured at 570 nm wavelength with a 650 nm reference wavelength using the Tecan Sunrise^®^ microtitre plate reader (Tecan, Switzerland). Assessment on the type of combination relationship was done using an isobologram analysis, while the degree of synergy was assessed based on calculated combination index (CI) values, where CI values of >1.0 implies antagonism, 1.0 implies additivity, and <1.0 implies synergistic type relationships between two drugs. All calculations were based upon the CI equation adapted from previous literature [28].

### Effects of ACA *in vivo*

Athymic nude mice (*Nu/Nu*) were obtained from Biolasco Taiwan Co. Ltd. and used for all human oral SCC tumor xenografts. Male nude mice 6-weeks-old, weighing 27 g to 30 g were used and fed *ad libitum* with sterilized food pellets and sterile water. Tumor induction was done by injecting suspensions of 100.0 μl HSC-4 cells (1 x 10^7^cells/ml) in 1x PBS subcutaneously (s.c.) at the lateral neck region of *Nu/Nu* mice using 25 gauge needles. Both ACA (1.9 μg/ml) and CDDP (8.0 μg/ml for combination or 35.0 μg/ml for standalone) were dissolved in 0.9% (w/v) sodium chloride solution and administered via s.c. locally at tumor induction sites once tumor reached above 100.0 mm^3^ in volume. Standalone and combination treatments were administered three times a week at two day intervals via *in situ* s.c. injections, and sterile PBS solutions were used as placebo controls. Tumor volumes were assessed by measuring length x width x height with a Traceable Digital Calliper (Fisher Scientific) every 7-days post-treatment, and net body weight minus weight of tumors were measured. All animal studies were conducted in specific pathogen free (SPF) facilities with HEPA filtered air provided by Genetic Improvement and Farm Technologies Pte. Ltd. (GIFT) and were in accordance with the guidelines for the Veterinary Surgeons Act 1974 and Animal Act 1953. Housing and husbandry management were conducted according to guidelines by Institute of Laboratory Animal Resources (ILAR), while termination of specimens was done using purified CO_2_ gas according to the American Veterinary Medical Association (AVMA) Guidelines on Euthanasia.

### Immunohistochemistry (IHC)

Paraffin-embedded tumor biopsies were harvested, fixed in 10% (v/v) neutral buffered formalin (NBF) and embedded in paraffin for IHC analyses. Removal of paraffin from tissue sections were done using xylol followed by rehydration in a graded alcohol series. Epitope retrieval was achieved by boiling the tissue sections in sodium citrate buffer (0.01 M, pH 6.0) for 10 min. Endogenous peroxidase activity was blocked using 3% (v/v) hydrogen peroxide and washed. All sections were blocked with TBST and 5% (v/v) normal goat serum for 1 h. IHC was performed using antibodies specific for NF-κB p65 (1:400), IκBα (1:50), phospho-IKKα/β (1:300), COX-2 (1:200) and cyclin D1 (1:25). SignalStain^®^ Boost IHC Detection Reagent (HRP, Mouse/Rabbit) were used for signal detection according to the manufacturer's protocol and further developed with DAB solution. Counter-staining was done using hematoxylin and embedded with DPX mounting medium. Images were captured using an inverted fluorescence microscope Nikon Eclipse TS 100 (Nikon Instruments, Japan) and quantified using the Nikon NIS-BR Element software (Nikon Instruments, Japan).

### Statistical analysis

Data from all experiments were presented as mean ± SEM. Student’s two-tailed *t*-test was used to determine the statistical significance of results with *p* ≤ 0.05 or *p* ≤ 0.10 in some *in vivo* experiments. Migration assay experiments were performed in triplicates and all data were also reported as mean ± SEM of four sub-sections per replicate. All global gene expression and *in vitro* drug combination experiments were carried out in triplicates. All *in vivo* data were calculated based on five replicates per treatment or placebo group.

## Results

### ACA induces apoptosis-mediated cell death and suppresses the proliferation and migration rate of oral SCC *in vitro*

**Figure 1:**
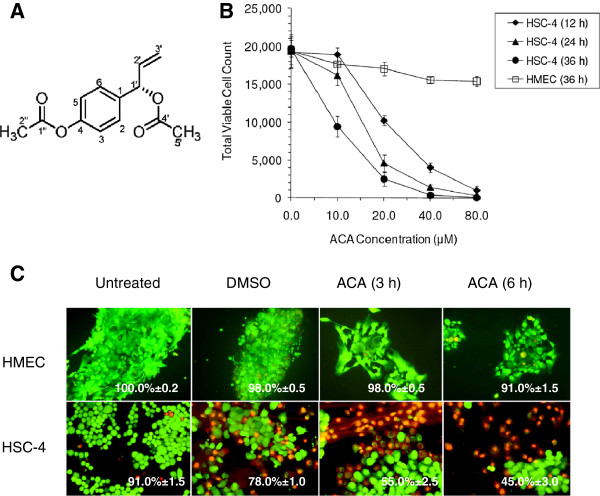
**(A) Chemical structure of 1’S-1’-acetoxychavicol acetate (ACA) from *Alpinia conchigera *Griff. (Zingiberaceae), Malaysian isolate.** (**B**) Comparison of total viable cell count of HSC-4 cells after treatment with ACA at different post-treatment time, indicating both time- and dose-dependent cytotoxicity. HMEC was used as a normal cell control. All MTT data were represented as mean ±SD of three independent experiments. (**C**) Live/Dead^®^ assay on HMEC normal cell controls and HSC-4 cancer cells upon treatment with ACA (15.0 μM) and DMSO solvent controls for 3 h and 6 h. Green fluorescence denotes viable cells stained with acetomethoxy derivate of calcein, while red fluorescence represents non-viable cells stained with ethidium homodimer-1. Relative mean percentages of viable cells are shown as calculated under a fluorescence microscope from three independent experiments. A total of four random quadrants were selected from each replicate for quantification.

**Figure 2:**
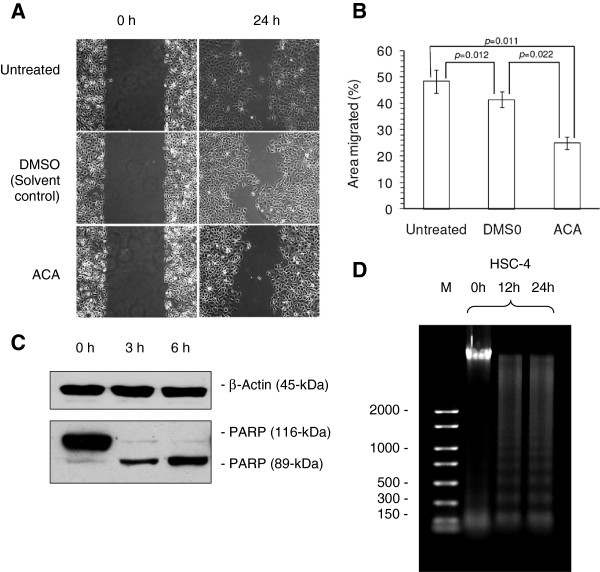
**The effects of ACA on the migration and apoptotic properties of HSC-4 human oral carcinoma cells.** (**A**) Inhibition on migration rate by ACA as demonstrated using the wound healing assay with DMSO as a solvent control. (**B**) Quantification on open wound areas after 24 h of incubation using the TScratch software. (**C**) Induction of apoptosis by ACA through activation of caspase-3 and subsequent cleavage of full length PARP enzymes (116 kDa) into a small (24 kDa) and a large (89 kDa) subunit protein, with β-actin as a loading control. (**D**) Confirmation of apoptosis-mediated cell death in HSC-4 cells through the visualization of 150 to 200 bps laddering of genomic DNA upon ACA treatment as demonstrated by DNA fragmentation assay.

As our previous studies have characterized the structure of ACA (Figure 1A) and confirmed it as a potent cytotoxic phytocompound on various cancer cell lines [18], the goal of this follow-up study was, first, to determine whether ACA could induce apoptosis-mediated cell death together with other anti-cancer properties such as anti-migration effects. To determine the effective cytotoxic dose of ACA, MTT viability assays and Live/Dead fluorescence assays were performed on HSC-4 oral SCCs at various incubation intervals over 36 h. Treatment with DMSO was used as a vehicle control to ensure the absence of solvent-induced cytotoxicity, while HMEC cells were used as a normal control to assess the effects of ACA on non-cancerous cells (Figure. 1B & 1C). IC_50_ values of ACA on HSC-4 cells fell within the range of 10 μM to 20 μM depending on various incubation periods, with minimal cytotoxic effects on HMEC cells where viability remained > 75% after a maximum of 36 h exposure. ACA was also found to reduce HSC-4 cell migration rates whereby the area of scratch wounds healed by 24.9 ± 2.3% compared to 48.3 ± 4.5% in untreated controls (*p*-value = 0.011) (Figure, 2A & 2B). The occurrence of apoptosis-mediated cell death was confirmed using PARP cleavage assays where full length PARP (116 kDa) was cleaved into a smaller 89 kDa fragment through caspase-3 activity (Figure 2C), while DNA fragmentation assays indicated a 150 kb to 200 kb laddering of DNA as early as 12 h upon ACA exposure, which is a strong hallmark of apoptotic events (Figure 2D).

### ACA dysregulated NF-ÎºB related genes as indicated through microarray global expression analysis

In order to assess cluster of genes affected upon exposure to ACA, a microarray global expression analysis was performed. Filtered gene expression data sets from HSC-4 cells treated with ACA for 1 h and 2 h were sorted based on top 20 genes related to proliferation, apoptosis and tumorigenesis that were up- and down-regulated as summarized in Table 1. A large portion of genes affected were found to be either directly or indirectly related to the NF-κB pathway, corresponding to 88% of the top 50 genes by fold change. Among the top up-regulated genes were those encoding p53, F-box proteins, cell cycle progression proteins and Bcl-2 family members. In terms of genes down-regulated by ACA, it was observed that a majority of these genes contained the κB binding sequence in its promoter region such as v-fos oncogene, Jun proto-oncogene, lymphotoxin-β, IκB-delta, TNF-R, TRAF-1 and TRADD (Table 1). As our microarray results were centered on the NF-κB pathway, we further investigated the direct relationship between ACA and various NF-κB family members using Western blot analysis.

**Table 1:** **Summary of the top 20 cancer and apoptosis-related gene expression changes in HSC-4 cells following ACA treatment for 1 h and 2 h. Genes were selected based on triplicates with*p*-values ≤ 0.05; and mean fold changes ≥ 1.50**

**Gene description**	**GenBank ID**	**p-value**	**Fold change (0 h vs. 1h)**	**Fold change (0 h vs. 2 h)**	**NF-kB relation**
Tumour protien p53	AK 125880	0.0090	2.01	1.91	Activator
F-box protien 21	BC034045	0.0009	1.95	1.73	Ubq.^b^
BCL2-associated X (Bax)	AK291076	0.0106	1.83	1.54	Gene Target^a^
Cyclin G2	BC032518	0.0216	1.74	1.65	Indirect Act.^c^
Tumour necrosis factor alpha (TNF-α)	BC064689	0.0411	1.62	1.63	Gene Target
SMAD 6	AF035528	0.0120	1.58	1.58	Ubq.
Endonuclease G	AK023235	0.0459	1.55	1.52	Gene Target
Poly (ADP-ribose) polymerase (PARP)	BC031073	0.0432	1.54	1.56	Activator
Caspase 6 (CASP-6)	U20536	0.0153	1.54	1.69	Indirect Act.
Cyclin-dependent kinase 2 (CDK-2)	U35146	0.0383	1.53	1.51	Indirect Act.
v-fos FBJ murine osteosarcoman viral oncogene	BX647104	0.0067	−8.53	−8.32	Activator
Jun oncogene	BC068522	0.0016	−3.32	−3.11	Activator
Fibroblast growth factor 2 (FBF-2)	J04513	0.0361	−1.84	−1.65	Gene Target
Lymphotoxin beta (LTB)	L11015	0.0429	−1.74	−1.58	Gene Target
Nuclear factor of kappa inhibitor delta (lkB-δ)	AF385434	0.0239	−1.65	−1.60	Inhibitor
Mitogen-activated protien 3-kinase (MAP3K)	BC035576	0.0313	−1.59	−1.59	Activator
Tumour necrosis factor receptor (TNF-R)	AF018253	0.0062	−1.56	−1.57	Gene Target
Tumour protien 73 (p73)	AY040827	0.0394	−1.56	−1.59	Indirect Act.
TNFR-associated death domain (TRADD)	BC004491	0.0421	−1.55	−1.55	Activator
TNF receptor-associated factor 1 (TRAF-1)	AK090468	0.039	−1.52	−1.55	Gene Target

a Gene Target: contains κB site in promoter region.

b Ubq: involved in ubiquitination process of IκBs.

c Indirect Act: gene is activated by another κB-regulated gene.

### ACA inhibits IKKÎ±/β-based phosphorylation and subsequent NF-ÎºB activation in HSC-4 cells

**Figure 3:**
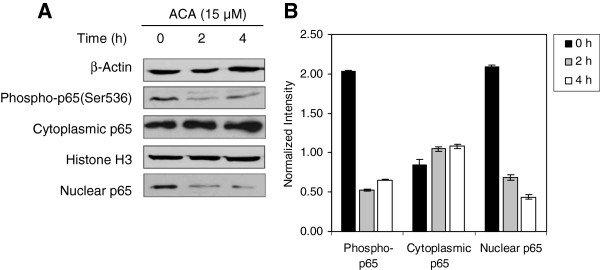
**ACA inhibits p65 (RelA) nuclear retention in HSC-4 human oral cancer cells.** (**A**) Western blot analysis on p65 localization levels indicated that cytoplasmic p65 increased, while nuclear p65 decreased upon ACA exposure for 4 h. (**B**) All band intensities between cytoplasmic and nuclear extracts were quantified and normalized against β-actin and histone H3 respectively, using the ImageJ v1.43u software.

**Figure 4:**
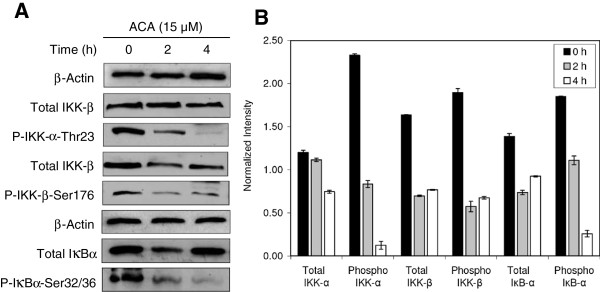
**ACA reduces NF-κB activation in HSC-4 human oral cancer cells.** (**A**) Reduction in IKK-α/β key phosphorylation sites prevents subsequent phosphorylation of IκB-α at Ser32/36, resulting in NF-κB inactivation. (**B**) All band intensities were quantified and normalized against β-actin using the ImageJ v1.43u software, and presented as mean ± SEM of three replicates.

Since the DNA binding capability of NF-κB transcription factors are governed by phosphorylation levels upon ubiquitination and subsequent release of IκBs from NF-κB heterodimers, Western blot analysis was conducted on both total and phosphorylated forms of p65, IκB-α/β proteins and the IKK complex. It was found that exposure to ACA reduced Ser536 phosphorylation levels of p65 subunits (Figure 3A & 3B), which suggested that ACA prevented C-terminal phosphorylation required by canonical NF-κB heterodimers for transactivation and commencement of κB gene transcription. Analysis of NF-κB heterodimer translocation between the nucleus and cytoplasm using antibodies against p65 (RelA) also revealed that levels of p65 within the cytoplasm increased corresponding with a reduction in nuclear p65 protein levels (Figure 3A & 3B). This consistently indicated that p65 nuclear localization was perturbed with an increasing ACA exposure time over 4 h. These observations corresponded with up-stream events which indicated that levels of phosphorylated IKKα on Thr23 and IKKβ on Ser176 were reduced, consistent with increasing ACA incubation periods (Figure 3A & 4B). Western blot assays on Ser32/36 phosphorylated forms of IκBα, which is a marker required for ubiquitination signalling, also indicated a reduction in band intensity, resulting in NF-κB heterodimers remaining within the cytoplasm (Figure 4A & 4B).

### ACA potentiates the cytotoxic effects of CDDP in HSC-4 SCC cells

**Figure 5:**
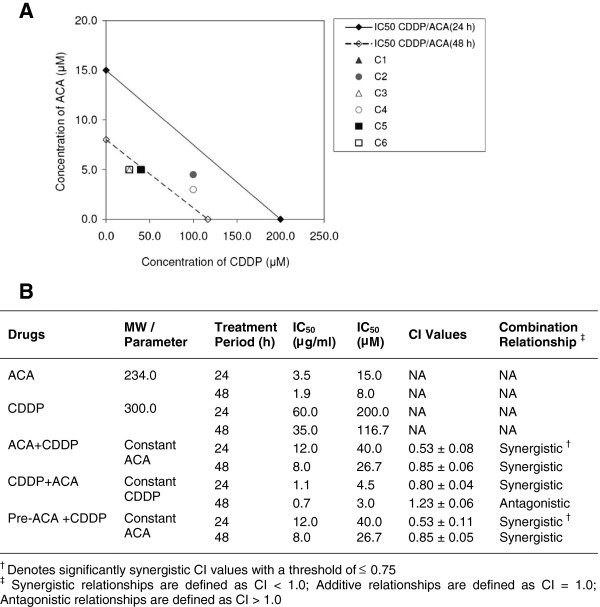
**Significantly transient synergistic relationships between ACA and CDDP, suggesting that ACA plays a role in HSC-4 chemo-sensitization.** (**A**) Isobologram IC_50_ analysis of HSC-4 cells after exposure to both ACA/CDDP simultaneous and sequential combinations over 48 h. C1: 24 h simultaneous treatment (constant ACA); C2: 24 h simultaneous treatment (constant CDDP); C3: 48 h simultaneous treatment (constant ACA); C4: 48 h simultaneous treatment (constant) CDDP; C5: 12 h pre-ACA prior to 24 h of CDDP sequential treatment; C6: 12 h pre-ACA prior to 48 h of CDDP sequential treatment. (**B**) Summary of IC_50_ and combination index (CI) values calculated from *in vitro* MTT cytotoxicity assays on various treatment combinations. Data were presented as mean ± SEM of independent triplicate experiments.

Since natural compounds that inhibited the NF-κB pathway are often sought for as sensitizing potentiators, we next investigated the combined synergistic *in vitro* anti-proliferative effects of ACA with CDDP in HSC-4 SCC cells. Based on MTT assay isobologram-illustrated results, it was found that ACA was able to reduce the viability levels of HSC-4 cells to a higher extent when used in combination with CDDP (Figure 5A). CI analysis indicates that synergistic effects (CI < 1.0) were observed for all combinations where ACA was maintained at a constant concentration and CDDP at varying concentrations for both 24 h and 48 h. However, as significantly synergistic CI value thresholds were set at CI < 0.75, only sequential regimes where cells were pre-treated with ACA for 12 h followed by CDDP for 24 h yielded synergistic drug interactions of CI = 0.53 ± 0.11. Regimes where ACA was held constant with varying CDDP also yielded similar CI values at 24 h, but was no longer synergistically significant at 48 h. Inversely, a shift towards an antagonistic effect (CI=1.23 ± 0.06) was observed when CDDP was maintained at a constant concentration, while ACA was allowed to vary after 48 h of incubation (Figure 5B). This concluded that ACA’s action through NF-κB down-regulation was successful in transiently sensitizing HSC-4 cells, hence, potentiating the cytotoxic effects of CDDP.

### ACA increases the efficacy of CDDP on HSC-4 oral SCC xenografts *in vivo*

**Figure 6:**
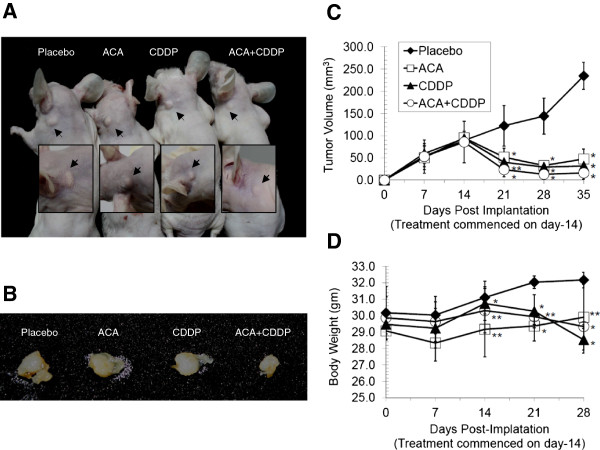
**Reduction of tumor volumes when treated with ACA and CDDP in combination on *Nu/Nu *mice.** (**A**) Photographs of *Nu/Nu* mice harvested 35 days post-implantation with human oral SCC (HSC-4) xenografts and 21 days post-treatment with various ACA/CDDP treatment regimes. Location of all tumor sites are indicated by closed arrows. (**B**) Representative photographs (*n*=5) of dissected oral tumors from different treatment regimes. (**C**) Tumor volume regression between various treatment groups 35 days post-implantation and 21 days post-treatment. (**D**) Assessment on body weight loss between various ACA/CDDP treatment groups. Data shown as mean value ± SEM of five replicates per group and 0.9% (w/v) sodium chloride solution was used in placebo groups. Statistically significant differences from placebo groups are shown by (*p ≤ 0.05) and (**p ≤ 0.1).

We next assessed the *in vivo* combined effects of ACA/CDDP on oral cancer xenografts in nude mice models. Following three weeks of a combined ACA/CDDP regime, where a lower CDDP dose (8.0 μg/ml) was used in comparison to a higher standalone CDDP dose (35.0 μg/ml), tumor volumes were found to be greatly reduced by 93.2 ± 5.2% compared to placebo groups. In reference to standalone groups, tumor volume reductions were 79.8 ± 9.5% and 86.5 ± 8.2% for ACA and CDDP respectively (Figure 6A to 6C), which corresponded to an efficacy improvement of 13.4% and 6.7% respectively in comparison to combined treatments. The use of ACA (1.9 μg/ml) to reduce effective CDDP dosage was also successful in reducing the extent of mean body weight loss by 2.5 ± 1.8% when compared to CDDP standalone treatments (Figure 6D). In addition, all mice appeared healthy during treatment, while histopathological analysis at autopsy revealed no ACA-induced tissue changes in any of the organs. Therefore, these results supported ACA’s potential to overcome dose-limiting toxicities brought upon by CDDP, while maintaining a similar, if not, higher efficacy level.

### ACA potentiates the efficacy of CDDP *in vivo* by down-regulating the NF-ÎºB pathway and NF-ÎºB regulated genes

**Figure 7:**
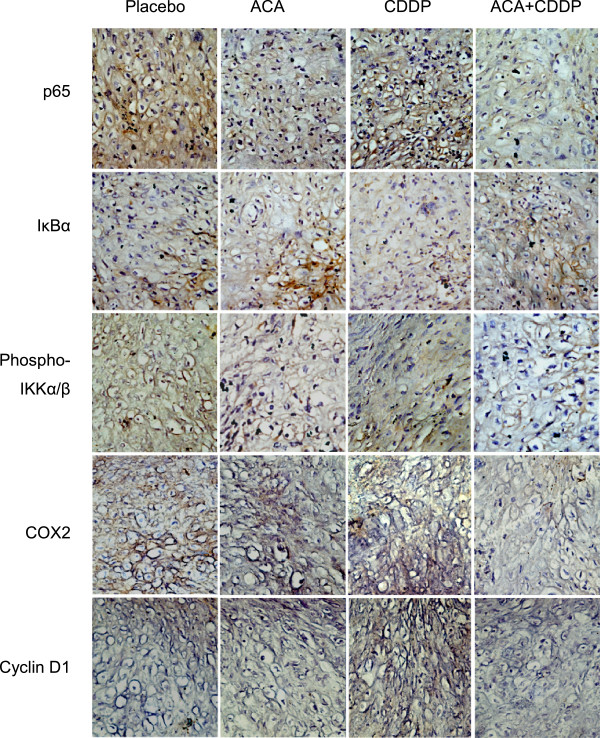
**Histopathological effects of ACA and CDDP treatment on HSC-4 human oral squamous carcinoma xenograft tumor biopsies against members of the NF-κB pathway and NF-κB regulated genes.** IHC analysis indicates an increase in IκBα protein levels and a reduction in p65, phospho-IKKα/β, COX-2 and cyclin D1 protein levels on sections treated with standalone ACA and ACA in combination with CDDP. Blue color indicates nuclei stained with hematoxylin and brown color indicates specific DAB antibody staining. All images were shown as a representative of three independent replicates at 400X magnification.

**Figure 8:**
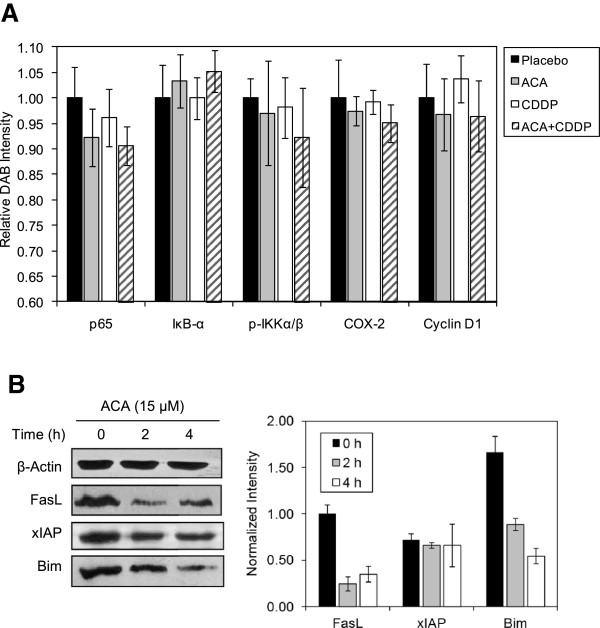
**ACA reduces the protein level of proinflammatory cytokines and other NF-κB regulated genes.** (**A**) Quantification of IHC DAB staining on HSC-4 human oral SCC xenograft sections treated with various ACA and CDDP combination regimes. Data for all NF-κB family proteins and NF-κB regulated proteins were presented as mean ± SEM of three independent replicates. (**B**) Western blotting indicating a reduction in FasL and Bim protein levels, while xIAP remains unaffected. All band intensities were quantified and normalized against β-actin using the ImageJ v1.43u software, and presented as mean ± SEM of three replicates.

To evaluate the consistency of ACA’s chemo-sensitizing and apoptosis-inducing effects *in vivo*, IHC assays were conducted on members of the NF-κB pathway as well as NF-κB regulated proteins. A reduction in p65 and phospho-IKKα/β protein levels were observed upon ACA treatment compared to placebo sections, but remained relatively similar after the incorporation of CDDP in ACA combination treatments (Figure 7 &8A). Subsequent effects on NF-κB regulated proinflammatory genes following alterations in NF-κB activation were also observed between placebo and ACA treated sections, indicating slight reductions in COX-2 and cyclin D1 protein levels. Protein levels of IκBα were also shown to increase in the presence of ACA, which were consistent with a reduction in IKK phosphorylation and subsequent prevention of 26S proteasomal degradation of IκBα as observed under *in vitro* conditions (Figure 7 &8A). Observation on other NF-κB regulated genes also indicate a reduction in Fas-ligand and Bim protein levels, however, xIAP levels remained unchanged following ACA treatment, thus suggesting the involvement of other post-transcriptional regulatory elements (Figure 8B).

## Discussion

In the current study, we demonstrated the natural ginger compound ACA’s ability to inhibit the growth of oral SCC cells alone and in combination with CDDP both *in vitro* and *in vivo.* Various natural compounds can and has been shown to sensitize cancer cells through various ways. For example, curcumin (diferuloymethane) has been shown to potentiate the apoptotic effects of chemotherapeutic agents such as gemcitabine and paclitaxel in human bladder cancer cells through the deactivation of the NF-κB pathway [9,10]. Recent reports have also shown that multi-targeted therapy has a higher success rate against cancer compared to mono-targeted therapies [29,30]. This newly emerging form of combination chemotherapy involving chemo-sensitizers and anti-cancer drugs have been gaining vast popularity among oncologist worldwide, whereby new combination regimes are continuously being developed to reduce drug resistance and with increased efficacies. Of note, our *in vivo* data have showed that ACA on its own or in combination with CDDP was able to reduce tumor volumes and toxicity levels, resulting in reduced body weight loss compared to CDDP on its own.

The activation extent of various signal transduction pathways involved in chemo-sensitivity such as the NF-κB pathway, explains how resistant or susceptible a cancer type is towards drugs [14]. Since activation of the NF-κB pathway also protects cells from undergoing apoptosis [31], it is theoretically viable that the successful blocking of this pathway would have a reverse effect on tumor cells through the induction of apoptosis and increased susceptibility towards other drugs. One of the early evidence describing this hypothesis was presented when studies on p65-deficient mice hepatocytes with an inactive NF-κB pathway was shown to induce massive levels of apoptosis [32]. Since then, there have been reports on various chemotherapeutic agents that were able to cause dysregulation of NF-κB and NF-κB target genes, leading to sensitization and apoptosis [12,33-35]. In addition to its anti-apoptotic role, NF-κB also induces cell proliferation and cell-cycle progression by regulating the expression of target genes including growth factors such as IL-2, COX-2 and cell-cycle regulators such as cyclin D1 [34,36]. Here, our IHC results has provided evidence indicating that ACA was not only able to down-regulate NF-κB activation, but also reduce the expression of NF-κB-regulated genes such as proinflammatory (NF-κB and COX-2) and proliferative (cyclin D1), which are up-regulated in most human oral neoplasia [37,38]. This was found to be a favourable observation based on past reports, where higher levels of cyclin D1 expression exhibited higher resistance to CDDP, and a reduction in its expression resulted in increased sensitivity [39].

Key regulatory steps in IKK activation involve phosphorylation of several sites on the catalytic IKKα/β subunit, as well as polyubiquitination-based activation of its NEMO subunit. Based on Figures 4 and 5, it was observed that ACA prevented the site-specific phosphorylation of IKKα/β at Thr23 and Ser176. This led to the assumption that ACA may either obstruct site-specific phosphorylation through a direct interaction with IKK, or modulate further upstream signalling kinases such as MEKK3, TAK1 and NIK [40,41]. Inactivation of the IKK complex in turn, prevented the phosphorylation of RelA/p50 bound IκB-α and its subsequent ubiquitination and degradation. The inability to remove of IκB-α from the heterodimer prevented RelA/p50 phosphorylation, and its localization within the nucleus, therefore inhibiting the canonical mode of NF-κB activation and the expression of downstream κB-regulated genes.

In normal cells, even though NF-κB is rarely constitutively expressed, with the exception of proliferating B cells, T cells, thymocytes, monocytes and astrocytes, basal levels of NF-κB expression still remains detectable [13]. Therefore, the incomplete inhibitory effects of ACA on the NF-κB pathway as shown in this study is ideal, since a complete shutdown will result in the loss of peripheral immunogenic properties linked to immunodeficiency symptoms, which will subsequently make ACA a non-viable drug candidate. Observations indicating that the chemo-sensitizing effects of ACA were momentary, with synergism diminishing after 24 h of exposure, suggested that phenylpropanoids such as ACA can be either metabolized or chemically modified within the cell to an unstable structure. This unstable state which does not accumulate within cells can also be viewed as a desirable trait of ACA, which in turn prevents a toxicity build-up within an *in vivo* system.

Despite current findings pointing towards the use of ACA as a viable drug candidate, several problem-arising issues such as solubility factors and the non-specific nature surrounding organic compounds such as ACA should be further investigated. However, they can be addressed through the manipulation of delivery methods to include soluble protein partners with tumor receptor specificity. Nevertheless, our results support further research on ACA to improve its shortcomings as well as its inclusion into clinical trials in patients with oral cancer.

## Conclusion

Overall, our studies have proven that ACA can inhibit the growth of human oral cancer and further potentiate the effect of standard (CDDP) treatment by modulation of proinflammatory microenvironment. The current preclinical data could form the basis for further clinical trials to improve the current standards of care for oral malignancies, and perhaps other malignancies, using this active component of Malaysian ginger with an overall improved efficacy coupled with a lower effective CDDP dose.

### Ethical statement

We declare that all *in vivo* experiments were approved by the University of Malaya ethical committee, and were reported according to the ARRIVE guidelines as set by the National Centre for the Replacement, Refinement and Reduction of Animal in Research (NC3Rs). Animal care including housing, husbandry and termination were in accordance with the Veterinary Surgeons Act 1974 and Animal Act 1953, and guidelines by the Institute of Laboratory Animal Resources (ILAR) and American Veterinary Medical Association (AVMA).

## Competing interests

The author(s) declare that they have no competing interests.

## Authors’ contribution

LLAI carried out writing of the manuscript and experimental procedures including cytotoxicity assays, apoptosis assays, microarray analysis, combination studies, Western blot analysis and *in vivo* studies. NMA performed writing of the manuscript and experimental procedures including migration assays, *in vivo* studies and IHC assays. HI and KA participated in the design of the study and editing of the manuscript. NHN conceived the entire study and participated in the editing of the manuscript. All authors read and approved the final manuscript.

## Pre-publication history

The pre-publication history for this paper can be accessed here:

http://www.biomedcentral.com/1472-6882/12/179/prepub
